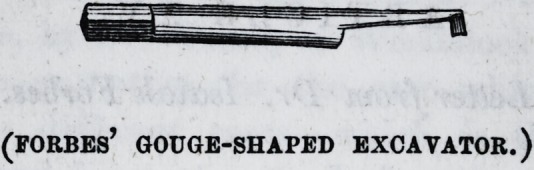# Letter from Dr. Isaiah Forbes

**Published:** 1859-04

**Authors:** I. Forbes


### ARTICLE XV.

### *Letter from Dr. Isaiah Forbes.*

Dr. Leslie :—

*Dear Sir* :—Having been frequently requested to give information as to the form, use and advantages of those curved instruments, which I have designated "Gouges" and also the V instrument, I beg permission to do so through the medium of your valuable journal.

1. *The Form.*—The shanks are all made sufficiently strong to prevent springing when under pressure. Some are straight, others bent at various angles, the same as ordinary excavators. "Gouges" are made of various sizes, from one to three lines in width, and from one to four lines in length; one with the face towards the left, another to the right, a third with the face towards the handle, and one out from it. The cutting edge of some form an arc of about one-third of a circle, others nearly one-half of a circle, always sharpening them on the face, thereby keeping the cutting edge on a line with the outer surface, so that the sides and end will cut when rotated. The end of the V instrument is formed by making the *acute* angle the longest side, and is principally used in cutting out the fissures.

With the "gouges" I cut away the enamel with more certainty, and excavate with greater facility and rapidity, preparing a large and difficult cavity with ease, and at the same time materially diminishing those disagreable gritting sensations which so often excite terror into the patient. I have used these instruments more than two years, and the more I use them the better I like them; and so satisfied am I of the advantages to be derived by their use both to me and the patient, that I would not be deprived of them for any consideration whatever.

Yours truly,

I. FORBES.

(FORBES' GOUGE-SHAPED EXCAVATOR.)

We have had the above cut of a small-sized "Forbes' Gouge" made to accompany Dr. Forbes' remarks, for the benefit of our readers. The rapidity with which, when necessary, sound dentine may be cut, will be understood by every one accustomed to the use of edge tools. The gouge cuts its piece clean out each stroke; it does not chip. The instrument above delineated, it will be understood, is to be used in the Forbes' socket, illustrated in our advertising pages.—[Ed. *Ib.*

## Figures and Tables

**Figure f1:**